# Extended time to maturity in *Anopheles coluzzii*: Implications of late egg hatch for vector control and transgene fitness

**DOI:** 10.1111/mve.12814

**Published:** 2025-06-06

**Authors:** Emmanuel C. Ottih, Joe M. Roberts, Toby J. A. Bruce, Frédéric Tripet

**Affiliations:** ^1^ Centre of Applied Entomology and Parasitology School of Life Sciences, Keele University Staffordshire UK; ^2^ Entomology Group Centre for Crop and Environmental Science, Harper Adams University Newport UK; ^3^ Swiss Tropical and Public Health Institute Allschwil Switzerland; ^4^ University of Basel Basel Switzerland

**Keywords:** *Anopheles coluzzii*, bidirectional selection, early and late hatching, genetically modified mosquitoes, Mopti, mosquito mass rearing, phenotypes, strains, VK

## Abstract

Maintaining fitness is an important consideration when mosquitoes are mass‐reared for the deployment of genetic interventions that are designed to suppress populations because released mosquitoes need to compete with wild‐type mosquitoes. Late‐hatching mosquitoes are more suitable for transportation to remote field sites. Here, we investigated the fitness of late‐hatching phenotypes in *Anopheles coluzzii*. Selected lines of the VK strain (from Burkina Faso) were created through bidirectional selection for early and late hatching, over 20 generations. These were compared with each other and the established Mopti reference strain from Mali, reared in the lab for >16 years. Significant differences in life‐history traits were found between Mopti and VK strains but few differences were found between the selected VK lines. Considering that late‐hatching VK lines showed no evidence of fitness costs, our results suggest that the late selected VK lines, which start hatching after 4 days, are an alternative option for egg shipment for mass mosquito releases over the well‐established Mopti that hatches within 2 days and has lower adult survival.

## Graphical abstract

- The fitness of *Anopheles coluzzii* mosquito lines (VK strain) selected for early and late egg hatching was compared with each other and a reference strain (Mopti).
- There was an observed increase in fitness in fecundity and survival among the late‐hatching lines, but no negative effects of selection on fecundity and adult survival were detected.
- This study informs that selected late‐hatching mosquito lines could serve as suitable starting lines for developing sterile or genetically modified lines intended for mosquito mass releases.

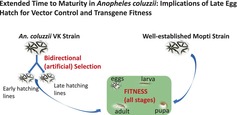

## INTRODUCTION

Malaria is a life‐threatening disease caused by the protozoan parasite *Plasmodium*, which is transmitted by infected female *Anopheles* mosquitoes. This disease continues to kill large numbers of people, with children under 5 years, pregnant women, travellers and immunocompromised individuals being at higher risk. In 2019, an estimated 227 million cases were reported worldwide and had risen to a total of 249 million cases by the year 2022 (World Health Organization, 2023). Insecticides are becoming less effective at controlling mosquito populations due to resistance, but the idea of using genetically modified (GM) mosquitoes to control this important disease vector is becoming more realistic (Favia, 2015; Yao et al., 2022).

The release of GM mosquitoes has been proposed for population replacement or population suppression by transgene technology (Alphey, 2014; Burt, 2014; Sinkins & Gould, 2006; Sougoufara et al., 2020). Population replacement systems, such as maternal‐effect dominant embryonic arrest (*Medea*), use a combination of toxins and antitoxin genes in the embryo that are suitable for replacing mosquito populations (Chen et al., 2007; Marshall & Akbari, 2016). Population suppression uses Clustered Regularly Interspaced Short Palindromic Repeat (CRISPR‐Cas9) to insert transgenes engineered to reduce the size of the wild population through a reduction in mosquito fecundity and introduce sex ratio distortion (Alphey, 2014; Burt, 2014; Xu et al., 2022). These techniques have been used on laboratory‐established anopheline populations (Galizi et al., 2014; Galizi et al., 2016; Hammond et al., 2016; Kyrou et al., 2018), as well as on *Aedes aegypti* for controlling Dengue and other diseases associated with the vector (Carvalho et al., 2015; Lacroix et al., 2012; Waltz, 2021).

GM mosquitoes have considerable potential as a novel vector control strategy and to complement existing control strategies, but success depends on ensuring the fitness of individuals. Individual fitness of an organism is considered to be the expected number of descendants it will leave in a given environment and depends on the survival and reproduction of the organism (Mills & Beatty, 1979). Understanding the disease vector's biology and life history, and ensuring the fitness of released mosquitoes, is necessary for effective control programmes using transgenic mosquitoes. Releasing mosquitoes to introduce the required transgene into a target population requires a population that can competently compete with its wild counterparts and, therefore, calls for effective evaluation of the fitness of the mosquitoes to be released. Even GM mosquitoes with gene drives need to be competitive at mating with wild mosquitoes for the initial introduction of the transgene into the wild population. Given the geographical distribution of the *Anopheles gambiae* complex, deploying GM mosquitoes would require shipping many eggs from production centres to egg‐to‐adult rearing units. *Anopheles gambiae* eggs generally hatch 2–3 days after oviposition, constraining the transportation of *Anopheles* eggs, particularly to remote locations. A solution to this could be the use of mosquito lines selected for late egg hatching (Ottih & Tripet, 2024). Understanding any impact of late hatching on fitness would help to assess the improvement of rearing and optimise performance.

The use of GM techniques for the control of malaria vectors would require a substantial number of mosquito eggs to be transported and distributed to local egg‐to‐adult rearing facilities in mass‐rearing programmes. In this context, one of the main constraints facing such programmes is that mosquitoes hatch in 2–3 days and shipping times to remote areas can take longer than this. To circumvent these limitations, environmental and genetic determinants of egg hatching were recently studied (Ottih & Tripet, 2024). Over 20 generations of early and late‐hatching strains were selected bidirectionally, opening the possibility of using delayed hatching to facilitate the shipping of eggs to remote mass‐rearing units. Early hatchers were defined as eggs that hatched within 2 days, while late hatchers were those that hatched between days 4 and 7.

While late hatching may be advantageous for transportation purposes, this needs to not incur a fitness cost. Mosquito fecundity and survival are important life‐history traits for adequate laboratory maintenance and mass releases. Recent studies have explored life‐history traits such as wing length, fecundity, survival, and insemination in different mosquito species (Pocquet et al., 2021; Yao et al., 2022). Since the genetic modification techniques rely on the release of genetically engineered males in large numbers, the efficiency of the techniques can only be evaluated through the assessment of male parameters such as longevity, dispersal ability and mating competitiveness (Diabate & Tripet, 2015). If the selected late‐hatching phenotypes were to be used as the starting genetic background for GM transformation, it is crucial that they have high survival and insemination success for ease of rearing in maintaining high survival and mating competitiveness for future releases. Bailey et al. (1979) reported high hatch rates in two species of *Anopheles* spp. after storing dry for 7 days, though life‐history traits such as longevity and mating competitiveness were not reported. However, results from a recent study, after measuring different parameters using fresh and dried eggs, suggest that drying *Anopheles* eggs could impact the longevity of the males, which is one of the important life‐history traits measured in mass releases of the male anopheline mosquitoes (Maiga et al., 2016).

In this study, the reproductive success and fitness of lines selected for late and early egg hatch and a long‐established reference strain of *An. coluzzii* were evaluated. Life‐history traits such as fecundity, wing length, insemination success and larval and adult survivorship were studied under laboratory conditions to explore the possible negative consequences of selection for different egg hatch times. Information obtained from this study will be useful for improving mosquito‐rearing protocols in laboratories. Our results inform whether selected late‐hatching strains, more amenable for egg shipment, are a viable choice as starting strains for sterile or GM strains to be used for mosquito mass releases.

## MATERIALS AND METHODS

### Strains and selection

The two strains of *Anopheles coluzzii* used in this study were the Mopti strain (Previously named *An. gambiae* “M” form) colonised in 2003 from N'Gabakoro Droit, Mali in West Africa (Baeshen et al., 2014) and a more recently colonised VK strain established from Vallee du Kou district, Burkina Faso in 2017. The VK strain was bidirectionally selected for early and late egg‐hatching phenotypes. Two pairs of lines (Late1, Late2, Early1 and Early2) were created through the bidirectional selection of the VK strain over 20 generations (Ottih & Tripet, 2024).

The early and late‐hatching lines tested originated from a common source colony and were subsequently established via bidirectional selection. Eggs from the parent generation were obtained by preparing 60 petri dishes (13 mm height by 60 mm diameter) holding 6–10 mL of mixed (mineral and deionised) water, with each dish holding 30 eggs. The petri dishes were agitated by spraying them with cold water (4 degrees) daily for 7 days, starting 1 h after preparing the eggs. The larvae that hatched on the second day were collected and considered the early hatchers, while larvae that hatched from day 4 to 7 were collected and labelled late hatchers. Larvae from both early and late‐hatching phenotypes were reared separately under the same conditions, with each providing two lines (early1 and early2 plus late1 and late2 hatchers) making up a total of four lines, with each line containing approximately 400 mosquitoes. These were combined into early and late hatchers for analysis. After establishing the first pair of early and late lines from the parental generation, the same procedure was repeated from the first generation of the selected phenotypes. For each of the selected lines, the larvae produced from early and late‐hatching eggs were used to establish the next generation. Larvae that hatched later than the second day in the fast‐hatching selected line were culled. For the selection of late‐hatching mosquitoes, any early emerging larvae (hatching in less than 2 days) were culled out to maintain strictly late hatchers. Early hatchers were defined as eggs that hatched within 2 days, while late hatchers were those that hatched between days 4 and 7.

### Mosquito rearing

Mosquitoes were maintained at a temperature of 26 ± 2°C and 75 ± 5% relative humidity (RH), under a photoperiod of 12:12 (light: dark) hours cycle. The larvae were reared at a density of 200 per tray (32.5‐inch L by 22‐inch W by 5‐inch H) and given Liquifry No1 fish food (2 drops, 120 μL) (INTERPET, Dorking, UK) on the first day, and were subsequently maintained on ground tropical fish food (Tetramin, Tetra, Melle, Germany). During pupation, collected pupae were transferred into a 100 mL polystyrene cup containing deionised water and placed in the centre of a cage for adults to emerge. On pupation, about 300–600 male and female pupae from each line (Mopti, VKEarly1, VKEarly2, VKLate1 and VKLate2) were aspirated into a polystyrene cup (50 mL). The emerged adults were placed in mosquito‐netted roof cages made of a 5‐litre white polypropylene bucket (19.5 cm height by 19 cm diameter), and an opening by the side with a netting sleeve through which a hand can access the interior of the cages. All adults were always provided with a 10% glucose solution.

### Egg collection

Three to four days after emergence, after mating had taken place, females were arm‐fed until they were fully engorged. On the third day after blood feeding, 30 gravid females were mouth aspirated into individual abraded specimen tubes containing 3–4 mL of deionised water, lined with a strip of filter paper, which served as an oviposition site. The females were allowed to lay eggs for 3 days and monitored daily from the day after the egg preparation. Eggs laid by each female were counted each day and recorded under a binocular microscope and resubmerged in water to hatch. Eggs laid by each female were counted and labelled as families to determine the fecundity of each female in the selected lines.

### Fitness assessment

The life‐history traits assessed and numbers of mosquitoes used are summarised in Table 1. The experiment assessing fitness ran with three replicates for each line, making up 15 replicates for the five lines treated and at their respective generations used (Early1: 21, 23, 25; Early2: 33, 38, 42; Late1: 19, 21, 23; Late2: 32, 34, 36 and Mopti).

**TABLE 1:** Number of mosquitoes used for each life‐history trait tested.

Life‐history trait	Egg count (Figure 1a)			Hatch rate (Figure 1b)			Pupation rate (Figure 1c)			Emergence rate (Figure 2b)			Insemination rate (Figure 3a)			Wing length (Figure 3c)		
Calculation	No. eggs laid			No. larvae/No. eggs			Total pupae/Total larvae			Total adults/Total pupae						Males and females		
Strains	R1	R2	R3	R1	R2	R3	R1	R2	R3	R1	R2	R3	R1	R2	R3	R1	R2	R3
Mopti	1492	2056	2792	539	1691	1539	459	1543	1323	434	1482	1249	120	120	120	120	120	120
Early1	768	2013	2019	441	1506	1537	403	1446	1420	386	1346	1284	120	120	120	120	120	120
Early2	2713	2333	1603	1455	1833	1349	1347	1643	1305	1308	1555	1244	120	120	120	120	120	120
Late1	1195	2045	1980	820	1395	1372	776	1204	1327	705	1098	1205	120	120	120	120	120	120
Late2	1840	1628	2401	1285	1355	2049	1118	1291	1850	1061	1218	1780	120	120	120	120	120	120

*Note*: ‘R’ represents repeats (batches). ‘Pupae emerged’ refers to the number of pupae used to assess adult longevity. The experiments for wing measurement and insemination were different setups: For wing measurement, 60 adults per sex (M or F) from each strain were measured per repeat, while for insemination, 120 females from each strain were used per repeat.

### Female fecundity

The wet filter papers containing eggs laid by an individual female were carefully lifted out of each tube using a pair of tissue forceps to ensure no eggs were lost. In a situation where eggs stuck to the tubes, a wet soft‐bristle brush was used to release them. The filter papers containing the eggs were immediately unfolded and placed on a binocular microscope stage where eggs were counted and recorded.

### Egg hatch rate

The counted eggs were immediately washed into a 100 mL cup of distilled water with filter papers folded into a cone shape and covered with a petri dish lid. For Mopti and Early1 and Early2, eggs were allowed to hatch into larvae for 3 days (by spraying 4°C cold water), while Late1 and Late2 strains were allowed to hatch for 7 days because few eggs started hatching from day 4. The emerged larvae were counted using a Pasteur pipette and recorded as they were transferred into rearing trays measuring (32.5‐inch L by 22‐inch W by 5‐inch H) to determine the egg hatch rate. Unhatched eggs were determined by subtracting hatched eggs from the total eggs laid. The rearing tray held 500 mL of mixed water (mineral and deionised) with 1 drop of liquid on the first day; an additional 500 mL of deionised water was subsequently added at a later stage (day 5). The amount of food fed to the individual family of the larvae varied and was determined by the number of larvae in the trays hatched per family. Larval survival was also determined by subtracting the total larvae that pupated from the total larvae hatched.

### Emergence success

Pupae were collected daily during the pupation period using an electric aspirator. The collected pupae were sexed under the dissecting microscope and counted to determine the sex ratio for each female in each line. After separating male and female pupae, the number was recorded and transferred into petri dishes (20 mm height by 50 mm diameter), and then into separate sex‐specific cages (polystyrene cups measuring 12 cm height by 11.3 cm diameter with netting tops and squared openings by the side covered with netting material). The pupae that emerged into adults were maintained in cages throughout their adult life until day 17. After emergence, dead pupae were counted and recorded by observing the petri dishes that contained the pupae to determine the emergence success from pupae to adults. The unmated adult males and females in separate cages were maintained with a 10% glucose solution using cotton wool, placed on top of the cage, and left under standard insectary conditions for 17 days. The 10% glucose solution was replaced ad libitum.

Dead adults were counted and removed with a pair of tissue forceps daily (24 h) for 17 days to determine the adult (male and female) longevity rate. The dead mosquitoes were continuously removed, counted and recorded until the last day of the experiment. On the 17th day, dead adults were counted and removed while the surviving ones were killed by being placed in the freezer after which they were counted.

### Insemination rate and dissection of spermatheca

Mosquitoes were reared and maintained as described above. The emerged pupae were sexed into males and females during pupation using a dissecting microscope. Only adult mosquitoes that emerged on the first day were used in the study to ensure all mosquitoes were of the same age.

The sexed males and females were transferred into 100 mL polystyrene cups and then placed into cages (5‐litre white polypropylene buckets measuring 19.5 cm in height by 19 cm diameter with a netting top and an opening by the side covered with netting material through which a hand can have access to the inside). The experiment was run in triplicate for each line (Early1 and Early2, Late1 and Late2 and unselected Mopti), with each repeat having three groups (cages) of 20 and 100, in the ratio of 1:5 male and female pupae respectively, which emerged into adults. The emerged adults were maintained with a 10% sugar solution in a 50 mL bottle with a paper towel folded into a wick. Two nights after emergence, 40 females were selected from each of the three groups, subsequently killed and stored in 80% ethanol for spermathecal dissection, to determine the insemination rate. Each of the females to be dissected was placed on a glass slide with a drop of water where the spermatheca was detached from each female using a needle. Spermathecae from the female mosquitoes were dissected in another drop of water on the same slide. The presence of a condensed sperm bundle confirms a successful mating taking place.

### Wing length

Wing length of both males and females was measured as a proxy for body size. Two days after emergence, 20 adult males and 20 adult females were selected from each of the three cages in each line to represent a population of each of the cages. From each group, the selected samples were killed in a 4°C fridge freezer and placed in 80% ethanol and kept in a −20°C freezer until ready to use. To determine wing length, each wing was removed from each sex using a pin and measured under a dissecting microscope. A binocular microscope calibrated using a stage unit micrometre (1 mm stage unit = 25 eyepiece units at 2.5 magnification) was used to measure one wing from each adult. Each wing was measured from the distal end of the alular notch to the distal end of the R2 wing vein in a drop of water on a glass microscope slide. Fringe hairs were excluded while measuring the wing length.

### Statistical analysis

All analyses were carried out in R (v4.4.1) using the following packages: lme4 (Bates et al., 2015), Car (Fox & Weisberg, 2019), emmeans (Lenth, 2024), MASS (Venables & Ripley, 2002), multcomp (Hothorn et al., 2008) and multcompView (Graves et al., 2024). Analyses were designed to compare the life‐history traits between the experimentally selected early and late phenotypes. This approach was selected because the primary research interest lies in understanding the potential fitness costs associated with these divergent phenotypes, rather than quantifying variation among the specific selection lines within each phenotype group.

#### Fecundity, egg hatch and larval survival

Fecundity (i.e., eggs per female) was initially analysed using a Poisson generalised linear mixed model (GLMM), the Pearson residuals indicated overdispersion, and so a negative binomial GLMM was used. The egg hatch rate was analysed as a binomial GLMM using the two‐column response [hatched, unhatched]. Larval survival (i.e., proportion of larvae surviving to pupation) was similarly analysed via a binomial GLMM with the two‐column response [pupae, larvae not surviving]. In both binomial models, exponentiated coefficients are interpreted as odds ratios (ORs). For each analysis, replicate was included as a random effect to account for non‐independence among families. Post hoc pairwise comparisons of estimated marginal means were carried out using a Sidak adjustment for multiple testing. Differences among strains were summarised by compact letter displays, and statistical significance was defined as *p* < 0.05.

#### Pupal duration, emergence and overall emergence success

Pupation timing (i.e., days to pupation) was analysed using a Poisson GLMM with replicate included as a random effect, and potential overdispersion was evaluated via Pearson residuals. Pupal emergence (i.e., proportion of pupae that eclosed as adults) and overall emergence success (i.e., proportion of eggs‐producing adults) were both analysed with binomial GLMMs using the two‐column response [success, failure]. Post hoc pairwise comparisons were carried out using a Sidak correction for multiple testing, and group differences were summarised using compact letter displays. Differences among strains were summarised by compact letter displays, and statistical significance was defined as *p* < 0.05.

#### Insemination success and wing length

Insemination success (i.e., presence of sperm in a female) was analysed with a binomial GLMM, including random intercepts for replicate, cage and female ID to account for nested variability. Wing length (mm) was compared among strains and between sexes using a Gaussian GLM. Model assumptions (e.g., normality and homogeneity of variance) were evaluated through residual diagnostics while post hoc pairwise comparisons were carried out using a Sidak correction. Statistical significance was defined as *p* < 0.05.

#### Adult survival

Adult survival was assessed by analysing the cumulative mortality counts at Day 17 post‐emergence. This specific timepoint corresponds to the standard duration adults are typically maintained for colony propagation under our laboratory‐rearing schedule before being discarded. Cumulative mortality was computed by summing daily deaths within each family. We initially fitted a Poisson generalised linear mixed‐effects model (GLMM) that included strain, sex and day as fixed effects and replicate as a random intercept. Overdispersion was assessed by Pearson residuals, but because the dispersion ratio exceeded acceptable thresholds (>1.5) a negative binomial GLMM was used. Significance of terms was evaluated via type III Wald X^2^ tests and post hoc pairwise comparisons for the strain‐by‐sex interaction using the Sidak adjustment. Time‐series patterns were visualised by computing the mean of the daily cumulative mortality counts (and 95% confidence intervals) for each strain–sex combination and plotting them as lines with shaded ribbons.

## RESULTS

### Fecundity, egg hatch and larval survival

**FIGURE 1:**
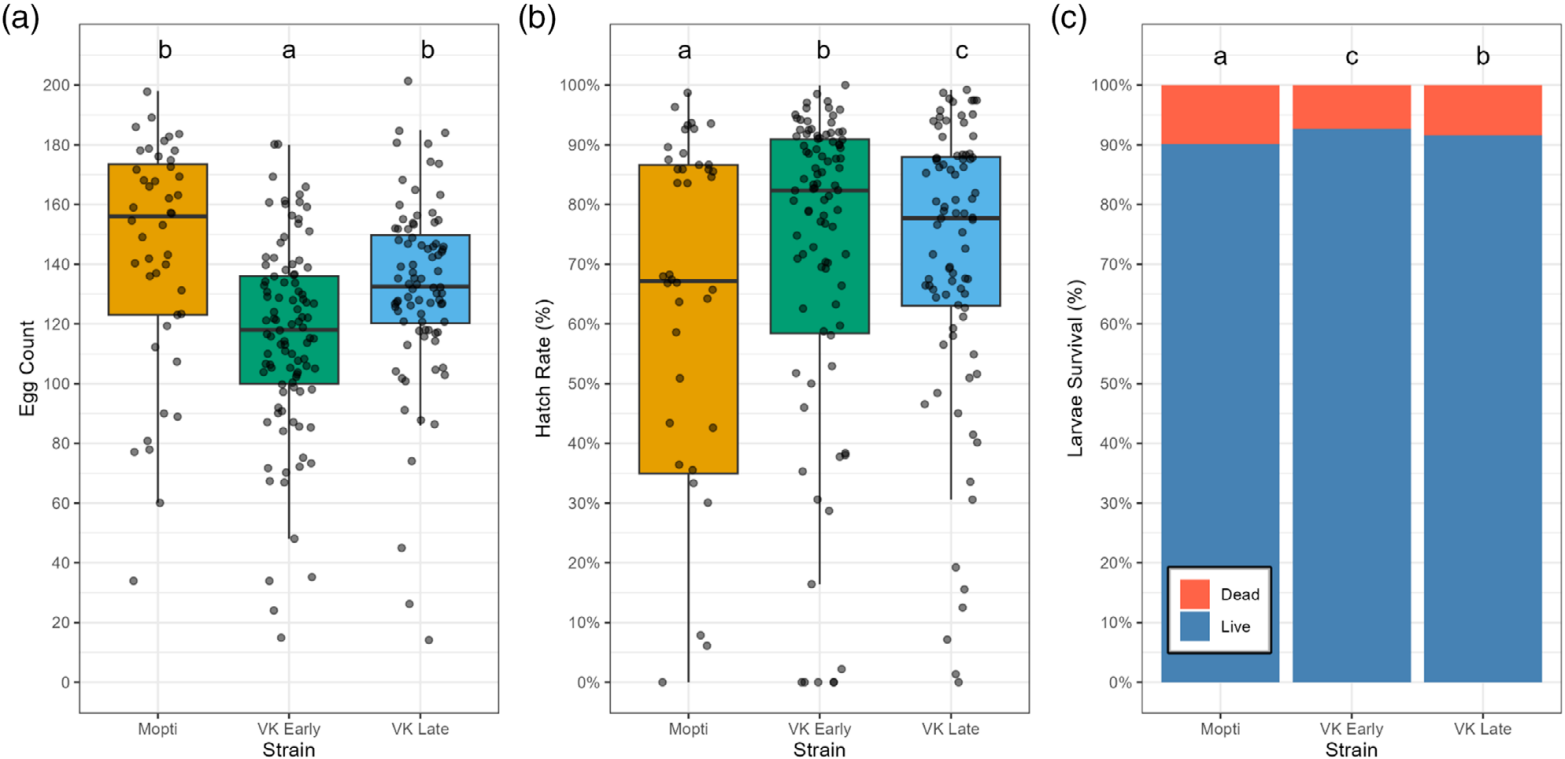
Reproductive performance in three mosquito strains: Mopti (orange), VK Early (green) and VK Late (blue). (a) Oviposition boxplots with individual data points. (b) Egg hatch rate boxplots with individual data points. (c) Larval survival (i.e., pupation rate) stacked bars of predicted survival vs. mortality. Boxplots depict medians and interquartile ranges, with individual data points overlaid. Letters above each strain denote significantly different groups (*p* < 0.05) based on negative binomial (egg counts) or binomial (hatch rate, survival) generalised linear mixed models with Sidak‐adjusted comparisons.

Fecundity varied significantly among strains, with Mopti ovipositing the greatest number, with a mean of 144 ± 6 (Figure 1a). Compared to Mopti, VK Early laid 20% fewer eggs (*z* = −3.93, *p* < 0.01), while VK Late showed an 8% reduction (*z* = −1.50, *p* = 0.135). Fecundity in VK Early was approximately 12% lower than in VK Late (*z* = −2.93, *p* = 0.0095) (Figure 1a). Egg hatch rates differed among strains, with Mopti showing the lowest hatch rate at 58% (Figure 1b). In contrast, VK Early and VK Late exhibited substantially higher hatch rates of 68% and 71%, respectively. Larval survival differed among strains with Mopti showing the lowest survival probability at 89% (*z* = 15.98, *p* < 0.01) (Figure 1c). Survival increased significantly in VK Early to 93% (*z* = 4.56, *p* = 0.01; OR = 1.38 [95% CI: 1.20–1.59]) and modestly in VK Late to 91% (*z* = 2.41, *p* = 0.0158; OR = 1.18 [95% CI: 1.03–1.35]). These represent absolute increases of 2.4% and 2.1% relative to Mopti.

### Pupal duration, pupal emergence and overall emergence success

**FIGURE 2:**
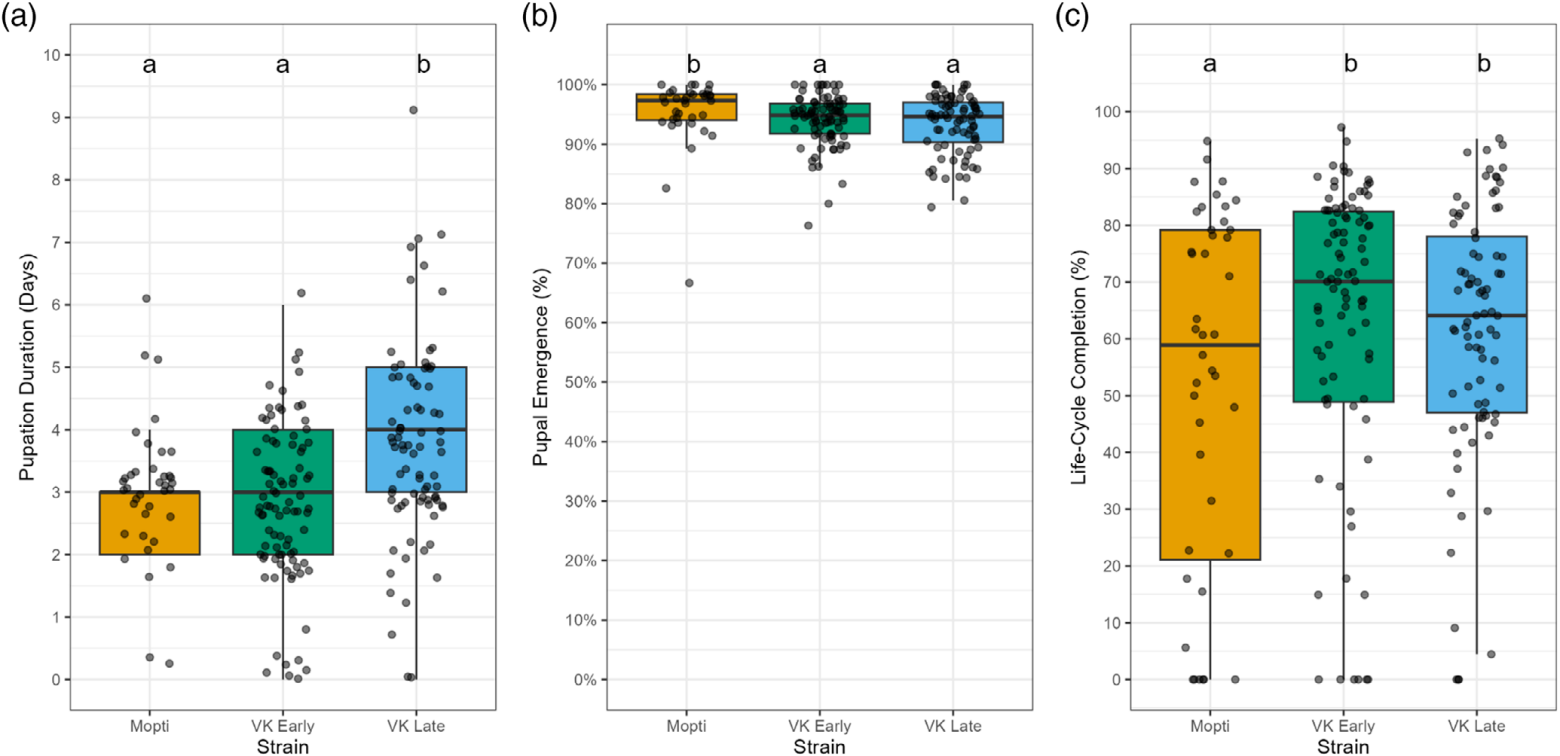
Pupal performance in three mosquito strains: Mopti (orange), VK Early (green) and VK Late (blue). (a) Pupation duration boxplots with individual data points (b) Pupal emergence boxplots with individual data points and (c) Life‐cycle completion boxplots with individual data points. Boxplots depict medians and interquartile ranges, with individual data points overlaid. Letters above each strain denote significantly different groups (*p* < 0.05) based on negative binomial generalised linear mixed models with Sidak‐adjusted comparisons.

Pupal duration varied significantly among strains. VK Late took significantly longer to pupate than both Mopti (*z* = 3.34, *p* < 0.01) and VK Early (*z* = 3.96, *p* < 0.01), whereas Mopti and VK Early did not differ (*z* = 0.29, *p* = 0.77). On average, Mopti and VK Early pupated in 3.14 and 3.05 days respectively, while VK Late required 3.86 days. The proportion of pupae successfully emerging as adults was highest in Mopti at 95% (*z* = −2.84, *p* = 0.0045), followed by VK Early at 94% (*z* = −4.23, *p* < 0.001) and VK Late at 93% (*z* = −4.23, *p* < 0.001) (Figure 2b). The difference between VK Early and VK Late was not significant (*z* = −1.89, *p* = 0.14). When measured as the proportion of eggs that developed into adults (i.e., life‐cycle completion or overall emergence), Mopti had a 51% emergence rate, whereas VK Early and VK Late reached 62% and 64% respectively (Figure 2c). Both VK Early (*z* = 15.05, *p* < 0.01) and VK Late (*z* = 16.81, *p* < 0.01) had significantly higher life‐cycle completion than Mopti. However, the difference between VK Early and VK Late was not significant (*z* = 2.11, *p* = 0.088).

### Insemination success and wing length

**FIGURE 3:**
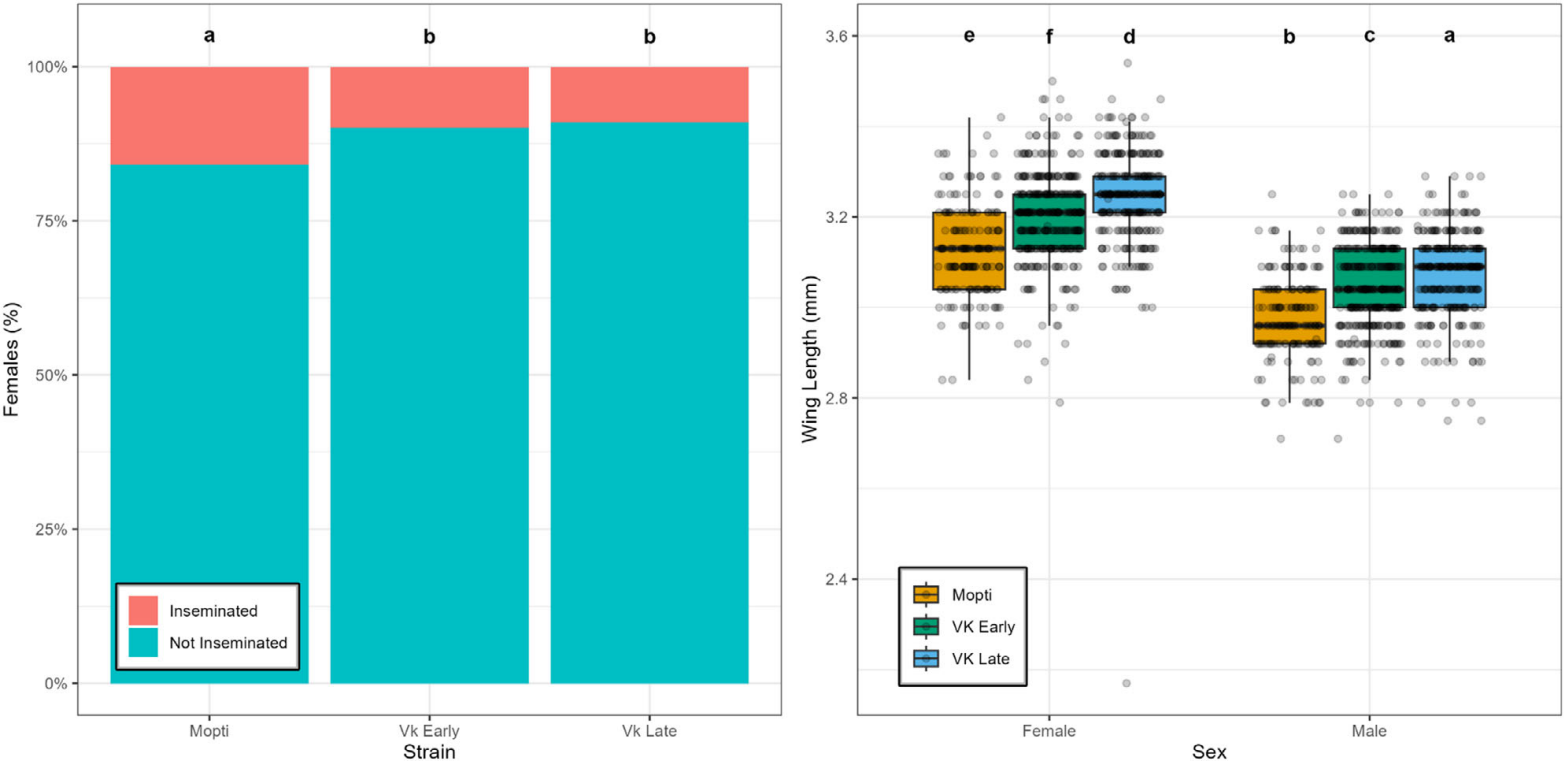
Reproductive success and wing morphology in three mosquito strains: Mopti (orange), VK Early (green) and VK Late (blue). (a) Female insemination success shown as a 100% stacked bar chart of inseminated (red) and non‐inseminated (teal) females. Insemination data were analysed using a logistic generalised linear mixed‐effects model (GLMM) with random effects for batch, cage and female ID. (b) Wing length (mm) by strain and sex shown as boxplots with overlaid individual data points. Wing length was analysed using a Gaussian linear model with additive effects of strain and sex. Boxplots show medians and interquartile ranges. Letters denote groups with significantly different marginal means (*p* < 0.05) based on Sidak‐adjusted pairwise comparisons.

Mopti exhibited a higher proportion of inseminated females compared to the other two strains (Figure 3a) at 15.8%. This is higher than VK Early (9.9%, *z* = −4.78, *p* < 0.001) and VK Late (9.0%, *z* = −5.37, *p* < 0.001). The difference between VK Early and VK Late was not significant (*z* = 0.91, *p* = 0.64). Wing length differed significantly by strain and sex (Figure 3b). Overall, females had longer wings than males (*t* = −33.61, *p* < 0.001). Among females, Mopti was the smallest (3.128 ± 0.006 mm), VK Early was intermediate (3.201 ± 0.004 mm) and VK Late was the largest (3.243 ± 0.005 mm). Among males, Mopti had the shortest wings (2.979 ± 0.006 mm), followed by VK Early (3.046 ± 0.004 mm) and VK Late (3.074 ± 0.005 mm).

### Adult survival

**FIGURE 4:**
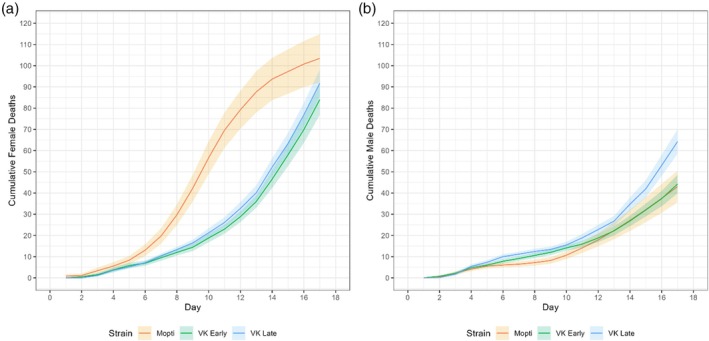
Cumulative mortality in three mosquito strains—Mopti (orange), VK Early (green) and VK Late (blue). (a) Female cumulative deaths over time. (b) Male cumulative deaths over time. Daily death counts were summed cumulatively within each replicate, and then averaged for each strain and sex. Plotted lines represent the mean cumulative deaths and the shaded ribbons represent 95% confidence intervals.

Strain and sex significantly influenced daily mortality (*p* < 0.001). Relative to Mopti females, VK Early and VK Late females had approximately 20%–25% lower daily mortality (*z* > 13.6, *p* < 0.001). Mopti males had about 67% lower mortality than Mopti females (*z* = −11.01, *p* < 0.001). Mortality increased by 17% per day (z = 30.87, *p* < 0.001), consistent with the upward trajectory in cumulative deaths over time (Figure 4). Mopti females exhibited the steepest rise in mortality, reaching approximately 104 total deaths by Day 17, whereas VK Early and VK Late females had fewer deaths by the end of the experiment, with 81 and 94, respectively. This pattern was reversed in males, where Mopti males showed the lowest mortality (44 deaths by Day 17), while VK Late males exhibited the highest mortality (64 deaths) and VK Early males had similar mortality levels to Mopti (44 deaths).

## DISCUSSION

This study set out to determine the fitness consequences of selecting for late‐hatching phenotypes in the malaria vector *An. coluzzii* over multiple generations, with a focus on key life‐history traits and their implications for both laboratory‐based rearing and field deployment of GM mosquitoes. The growing need for innovative and effective mosquito control strategies has led to increased attention on gene drives and other population‐suppression technologies that rely on the mass release of large numbers of mosquitoes (Alphey, 2014; Burt, 2014; Sinkins & Gould, 2006). Achieving success in these control measures requires careful balancing of several factors, including high productivity in the insectary, logistical feasibility of transporting mosquito eggs or larvae, and the competitiveness of released mosquitoes in the wild (Scott et al., 2002; Sougoufara et al., 2020). Thus, understanding any fitness trade‐offs associated with distinct phenotypes, such as differences in egg‐hatching time, holds significant importance for optimising genetic control programs.

Our findings demonstrate that late‐hatching lines do not exhibit a significant fitness penalty when maintained under standard insectary conditions. Indeed, we observed that most key reproductive indicators remained generally comparable or sometimes showed modest improvements in comparison to the Mopti strain. This aligns with a broader view of fertility as a multifaceted trait where robust insemination rates underpin high egg production and strong hatch rates confirm that those eggs are viable. Delayed hatching does not appear to undermine the capacity of females to produce and fertilise eggs effectively. We interpret this as evidence that selecting for delayed hatching does not inherently undermine the reproductive output or survivorship of *An. coluzzii* within the confines of a laboratory environment. In many large‐scale release programmes, one of the primary logistical challenges is ensuring that eggs can be transported to distant rearing or release sites without hatching prematurely; late‐hatching lines mitigate this risk by remaining quiescent longer, potentially enabling more efficient storage and transport (Maiga et al., 2016; Ottih & Tripet, 2024). This aspect of the late‐hatching phenotype could be especially beneficial in regions where infrastructure is underdeveloped and where unpredictable shipping durations might jeopardise egg viability.

Nevertheless, assessing the potential implications of delayed hatching on adult mortality proved critical to fully ascertain whether a lengthened egg stage might negatively affect subsequent developmental stages. In many genetic control programmes, especially those relying on gene drive systems or transgenic males for population suppression, adult longevity is a key parameter (Burt, 2014; Kyrou et al., 2018). High survival rates in insectary colonies ensure that sufficient adult numbers can be produced, while also supporting robust breeding and colony maintenance. Had there been a pronounced decrease in adult survival linked to the late‐hatching phenotype, the practicality of adopting such lines for mass‐rearing operations would have been seriously called into question. Our data indicated that adult survival trajectories, monitored up to 17 days post‐emergence, did not differ significantly between late and early hatching lines but did differ from Mopti. Both phenotypes exhibited similar mortality patterns, suggesting that shifting the hatch window later does not confer a significant survival disadvantage under controlled insectary conditions.

Field deployment considerations add another layer of complexity. Adult survival in natural settings is often considerably shorter than in laboratories, primarily due to environmental stressors, predation and resource limitations (Lyimo & Takken, 1993). Many studies estimate that the average adult female *An. coluzzii* survives approximately 2 weeks or less in the wild, depending on seasonal conditions, the presence of predators, and the availability of blood meals (Ameneshewa & Service, 1996; Lyimo & Takken, 1993). Importantly, since the late‐hatching lines in our study lived as long as early hatching lines under optimal conditions for more than 2 weeks, it is reasonable to speculate that these lines would not suffer acute survival deficits when faced with typical field conditions. This is a crucial point for any gene drive initiative: to effectively introduce or spread a transgene within a wild population, released mosquitoes must not only survive to mate, but also remain healthy enough to engage in multiple reproductive cycles if needed (Xu et al., 2022). Should late‐hatching strains exhibit compromised adult survival in the wild, the utility of their delayed egg‐hatching trait would be overshadowed by poor mating success or reduced transgene propagation.

Delayed hatching does introduce a potential developmental trade‐off that extends from the egg to the adult stage. By taking an additional 3–4 days to hatch, these mosquitoes effectively increase the time until they reach sexual maturity, which could diminish the number of generations achievable within a given season. From a multi‐generation perspective, this means that in highly seasonal or competitive environments, a strain that matures more slowly may experience reduced fitness if faster‐cycling wild strains complete additional reproductive rounds in the same timeframe. Consequently, even if we detect no direct penalty in single‐generation measures of fitness, such as fecundity or survival, a longer developmental window might lower the cumulative reproductive rate of a late‐hatching line over multiple generations. In other words, there may be a fitness cost in the broader population dynamics sense, even if not apparent through short‐term metrics.

When weighing the merits of late‐hatching phenotypes, it is important to consider the broader ecology and logistical demands of the release programme. For certain mass‐rearing initiatives, the ability to ship eggs reliably over long distances without unplanned hatching might represent a tangible advantage that outweighs the potential downsides of slower generational turnover once in the field (Maiga et al., 2016). Moreover, where *Anopheles* spp. populations face intense environmental pressures, such as insecticide exposure or predation (Sougoufara et al., 2020), the benefits of flexible hatching times may prove context‐dependent. It is also noteworthy that in large‐scale operations, synchronisation of egg‐to‐adult schedules can streamline rearing, which may synergise with the predictably delayed hatch of late‐hatching strains. Assessing the interplay of these factors in various ecological and infrastructural settings will be key to determining whether the late‐hatching trait is uniformly advantageous or if it must be tailored to specific release strategies.

Our study suggests that selecting for a delayed hatch trait within *An. coluzzii* does not produce overt detriments to key single‐generation life‐history traits. In fact, for mass‐release operations requiring eggs that remain viable over extended shipping periods, late‐hatching lines may enhance operational efficiency (Maiga et al., 2016; Ottih & Tripet, 2024). Beyond these immediate considerations, the potential effect of delayed hatching on multi‐generation population dynamics should not be underestimated. It remains possible that slower development might diminish the rate at which a gene drive or sterile insect technique can spread through a given population (Alphey, 2014; Burt, 2014). Future investigations should seek to measure these lines' long‐term competitiveness in semi‐field enclosures or field trials, where the full scope of environmental challenges can manifest. Additionally, measuring alternative fitness metrics, like male mating vigour under crowded or resource‐limited conditions or the capacity of females to complete multiple gonotrophic cycles under cyclical feeding availability, would further clarify whether late hatching confers any hidden disadvantages (Yao et al., 2022).

## CONCLUSION

Our findings confirm that artificially selecting for late‐hatching phenotypes in *An. coluzzii* can deliver tangible benefits for mass‐rearing and transport logistics, as eggs remain quiescent for longer periods, reducing the risk of premature hatching. At the same time, adult survival, fecundity, hatch rates and insemination success remain broadly similar to the original strains, indicating that single‐generation fitness does not appear to be compromised by delayed hatching. From a practical standpoint, these traits suggest that late‐hatching lines could be used to expand the geographical reach of GM mosquito release programs. However, their slightly extended developmental time may reduce the number of mosquito generations per year, which could, in turn, slow the spread or persistence of transgenes or other genetic control mechanisms. Ultimately, the relative advantages of enhanced shipping and storage capability must be balanced against this potential multi‐generation trade‐off. Further investigation, especially under semi‐field or field conditions, is required to determine whether delayed‐hatching strains can achieve sustained performance and successfully integrate transgenes into wild populations. Such insights will be critical for designing, monitoring and scaling genetic vector control initiatives that make use of delayed‐hatching lines.

## AUTHOR CONTRIBUTIONS

**Emmanuel C. Ottih:** Conceptualization; investigation; methodology; data curation; validation; visualization; writing – original draft; writing – review and editing; formal analysis; project administration. **Joe M. Roberts:** Methodology; formal analysis; data curation; software; validation; visualization; writing – review and editing. **Toby J. A. Bruce:** Writing – review and editing; project administration; supervision; validation; visualization; conceptualization. **Frédéric Tripet:** Conceptualization; funding acquisition; methodology; project administration; resources; supervision.

## CONFLICT OF INTEREST STATEMENT

The authors declare no conflicts of interest.

## Data Availability

The data that support the findings of this study are openly available in Figshare at https://figshare.com/articles/dataset/Selecting_late_hatching_phenotypes_does_not_negatively_impact_the_fitness_of_Anopheles_coluzzii_strains/28505141/1 (Roberts, 2025). [Correction added on 20 June 2025 after first online publication: The Data Availability Statement has been updated.]

## References

[mve12814-bib-0001] Alphey, L. (2014) Genetic control of mosquitoes. Annual Review of Entomology, 59, 205–224. 10.1146/annurev-ento-011613-162002 24160434

[mve12814-bib-0002] Ameneshewa, B. & Service, M.W. (1996) The relationship between female body size and survival rate of the malaria vector *Anopheles arabiensis* in Ethiopia. Medical and Veterinary Entomology, 10(2), 170–172. 8744710 10.1111/j.1365-2915.1996.tb00724.x

[mve12814-bib-0003] Baeshen, R. , Ekechukwu, N.E. , Toure, M. , Paton, D. , Coulibaly, M. , Traoré, S.F. et al. (2014) Differential effects of inbreeding and selection on male reproductive phenotype associated with the colonization and laboratory maintenance of *Anopheles gambiae* . Malaria Journal, 13, 19. 24418094 10.1186/1475-2875-13-19 PMC3896703

[mve12814-bib-0004] Bailey, D.L. , Thomas, J.A. , Munroe, W.L. & Dame, D.A. (1979) Viability of eggs of *Anopheles albimanus* and *Anopheles quadrimaculatus* when dried and stored at various temperatures. Mosquito News, 39, 113–116.

[mve12814-bib-0005] Bates, D. , Maechler, M. , Bolker, B. & Walker, S. (2015) Fitting linear mixed‐effects models using lme4. Journal of Statistical Software, 67(1), 1–48.

[mve12814-bib-0006] Burt, A. (2014) Heritable strategies for controlling insect vectors of disease. Philosophical Transactions of the Royal Society of London. Series B, Biological Sciences, 369(1645), 1–8. 10.1098/rstb.2013.0432 PMC4024225 24821918

[mve12814-bib-0007] Carvalho, D.O. , McKemey, A.R. , Garziera, L. , Lacroix, R. , Donnelly, C.A. , Alphey, L. et al. (2015) Suppression of a field population of *Aedes aegypti* in Brazil by sustained release of transgenic male mosquitoes. PLoS Neglected Tropical Diseases, 9(7), e0003864. 26135160 10.1371/journal.pntd.0003864 PMC4489809

[mve12814-bib-0008] Chen, C.‐H. , Huang, H. , Ward, C.M. , Su, J.T. , Schaeffer, L.V. , Guo, M. et al. (2007) A synthetic maternal‐effect selfish genetic element drives population replacement in *Drosophila* . Science, 316, 597–600. 17395794 10.1126/science.1138595

[mve12814-bib-0009] Diabate, A. & Tripet, F. (2015) Targeting male mosquito mating behaviour for malaria control. Parasites & Vectors, 8(1), 1–13. 26113015 10.1186/s13071-015-0961-8 PMC4485859

[mve12814-bib-0010] Favia, G. (2015) Engineered mosquitoes to fight mosquito‐borne disease: not a merely technical issue. Bioengineered, 6(1), 5–7. 25495663 10.4161/21655979.2014.988556 PMC4601486

[mve12814-bib-0011] Fox, J. & Weisberg, S. (2019) An R companion to applied regression, 3rd edition. Thousand Oaks CA: Sage.

[mve12814-bib-0012] Galizi, R. , Doyle, L.A. , Menichelli, M. , Bernardini, F. , Deredec, A. , Burt, A. et al. (2014) A synthetic sex ratio distortion system for the control of the human malaria mosquito. Nature Communications, 5, 3977. 10.1038/ncomms4977 PMC4057611 24915045

[mve12814-bib-0013] Galizi, R. , Hammond, A. , Kyrou, K. , Taxiarchi, C. , Bernardini, F. , O'Loughlin, S.M. et al. (2016) A CRISPR‐Cas9 sex‐ratio distortion system for genetic control. Scientific Reports, 6, 31139. 27484623 10.1038/srep31139 PMC4971495

[mve12814-bib-0014] Graves, S. , Piepho, H. & Dorai‐Raj, L.S.S. (2024) multcompView: visualizations of paired comparisons. R package version 0.1–10. Available from: https://CRAN.R-project.org/package=multcompView.

[mve12814-bib-0015] Hammond, A. , Galizi, R. , Kyrou, K. , Simoni, A. , Siniscalchi, C. , Katsanos, D. et al. (2016) A CRISPR‐Cas9 gene drive system targeting female reproduction in the malaria mosquito vector *Anopheles gambiae* . Nature Biotechnology, 34, 78–83. 10.1038/nbt.3439 PMC4913862 26641531

[mve12814-bib-0016] Hothorn, T. , Bretz, F. & Westfall, P. (2008) Simultaneous inference in general parametric models. Biometrical Journal, 50(3), 346–363. 18481363 10.1002/bimj.200810425

[mve12814-bib-0017] Kyrou, K. , Hammond, A.M. , Galizi, R. , Kranjc, N. , Burt, A. , Beaghton, A.K. et al. (2018) A CRISPR‐Cas9 gene drive targeting *doublesex* causes complete population suppression in caged *Anopheles gambiae* mosquitoes. Nature Biotechnology, 36, 1062–1066. 10.1038/nbt.4245 PMC6871539 30247490

[mve12814-bib-0018] Lacroix, R. , McKemey, A.R. , Raduan, N. , Wee, K.L. , Ming, W.H. , Ney, T.G. et al. (2012) Open field release of genetically engineered sterile male *Aedes aegypti* in Malaysia. PLoS One, 7, 1–9. 10.1371/journal.pone.0042771 PMC3428326 22970102

[mve12814-bib-0019] Lenth, R. (2024) emmeans: estimated marginal means, aka least‐squares means. R package version 1.10.3. Available from: https://CRAN.R-project.org/package=emmeans.

[mve12814-bib-0020] Lyimo, E.O. & Takken, W. (1993) Effects of adult body size on fecundity and the pre‐ gravid rate of *Anopheles gambiae* females in Tanzania. Medical and Veterinary Entomology, 7, 328–332. 8268486 10.1111/j.1365-2915.1993.tb00700.x

[mve12814-bib-0021] Maiga, H. , Damiens, D. , Diabate, A. , Dabire, K.R. , Quedraogo, G.A. , Lees, R.S. et al. (2016) Large‐scale *Anopheles arabiensis* egg quantification methods for mass rearing operations. Malaria Journal, 15(72), 1–7. 26852018 10.1186/s12936-016-1119-7 PMC4744385

[mve12814-bib-0022] Marshall, J.M. & Akbari, O.S. (2016) Chapter 9‐gene drive strategies for population replacement. In: Genetic control of malaria and dengue. Massachusetts: Cambridge, pp. 169–200.

[mve12814-bib-0023] Mills, S.K. & Beatty, J.H. (1979) The propensity interpretation of fitness. Philosophy of Science, 46, 263–286.

[mve12814-bib-0024] Ottih, E.C. & Tripet, F. (2024) Natural variation in timing of egg hatching, response to water agitation, and bidirectional selection of early and late hatching strains of the malaria mosquito *Anopheles gambiae* sensu lato. Parasites & Vectors, 17, 478. 39568044 10.1186/s13071-024-06533-w PMC11577942

[mve12814-bib-0025] Pocquet, N. , O'Connor, O. , Flores, H.A. , Tutagata, J. , Pol, M. , Hooker, D.J. et al. (2021) Assessment of fitness and vector competence of a New Caledonia wMel *Aedes aegypti* strain before field‐release. PLoS Neglected Tropical Diseases, 15(9), e0009752. 34492017 10.1371/journal.pntd.0009752 PMC8448375

[mve12814-bib-0111] Roberts, J. (2025). Selecting late hatching phenotypes does not negatively impact the fitness of *Anopheles coluzzii* strains. figshare. Dataset. 10.6084/m9.figshare.28505141.v1

[mve12814-bib-0026] Scott, T.W. , Takken, W. , Knols, B.G.J. & Boete, C. (2002) The ecology of genetically modified mosquitoes. Science, 298(5591), 117–119. 12364785 10.1126/science.298.5591.117

[mve12814-bib-0027] Sinkins, S.P. & Gould, F. (2006) Gene drive systems for insect disease vectors. Nature Reviews Genetics, 7, 427–435. 10.1038/nrg1870 16682981

[mve12814-bib-0028] Sougoufara, S. , Ottih, E.C. & Tripet, F. (2020) The need for new vector control approaches targeting outdoor biting anopheline malaria vector communities. Parasites & Vectors, 13, 295. 32522290 10.1186/s13071-020-04170-7 PMC7285743

[mve12814-bib-0029] Venables, W.N. & Ripley, B.D. (2002) Modern applied statistics with S, 4th edition. New York: Springer.

[mve12814-bib-0030] Waltz, E. (2021) First genetically modified mosquitoes released in the United States. Nature, 593, 175–176. 33976429 10.1038/d41586-021-01186-6

[mve12814-bib-0031] World Health Organization . (2023) World malaria report 2021. Geneva: WHO. Available from: https://www.who.int/teams/global-malaria-programme/reports/world-malaria-report-2023.

[mve12814-bib-0032] Xu, X. , Chen, J. , Du, X. , Yao, L. & Wang, Y. (2022) Crispr has also been used to mediate disruption of seminal fluid protein Sfp62 induces sterility in males in *Bombyx mori* . Biology, 11(4), 56. 10.3390/biology11040561 PMC9024854 35453761

[mve12814-bib-0033] Yao, F.A. , Millogo, A. , Epopa, P.S. , North, A. , Noulin, F. , Dao, K. et al. (2022) Mark release‐recapture experiment in Burkina Faso demonstrates reduced fitness and dispersal of genetically modified sterile malaria mosquitoes. Nature Communications, 13, 796. Available from: 10.1038/s41467-022-28419-0 PMC8831579 35145082

